# Association between Vascular Endothelial Growth Factor Plasma Levels and rs699947 Polymorphism and Coronary Collateral Vessel Formation 

**Published:** 2019-07

**Authors:** Mohammad Alidoosti, Mehrnoosh Shanaki, Armita Mahdavi, Narges Mohammadtaghvaei

**Affiliations:** 1 *Tehran Heart Center, Tehran University of Medical Sciences, Tehran, Iran. *; 2 *Department of Medical Laboratory Sciences, School of Allied Medical Sciences, Shahid Beheshti University of Medical Sciences, Tehran, Iran.*; 3 *Cellular and Molecular Research Center, Ahvaz Jundishapur University of Medical Sciences, Ahvaz, Iran.*

**Keywords:** *Vascular endothelial growth factor*, *Collateral circulation*, *Coronary vessels*, *Polymorphism*, *genetic*, *Coronary artery disease*

## Abstract

**Background:** The vascular endothelial growth factor (VEGF), as an angiogenic cytokine, binds endothelial cell receptors and stimulates angiogenesis and collateral formation. We evaluated the association between VEGF plasma levels and the gene polymorphism rs699947 and the formation of coronary collaterals in patients with coronary artery disease.

**Methods:** A total of 195 patients with ≥70% narrowing in at least 1 coronary vessel (according to coronary angiography) were included in the study. The presence of the rs699947 polymorphism within the promoter of the VEGF gene was investigated using polymerase chain reaction-restriction fragment length polymorphism (PCR-RFLP). The plasma VEGF concentration was quantified via the ELISA method. The Rentrop method was used to grade the extent of collateral development.

**Results:** There was no significant difference in VEGF levels between the groups with good and poor collaterals. The frequency of the A allele of rs699947 was found to be higher in the patients with good collaterals than in those with poor collaterals (P=0.014). The odds ratio of good collaterals for AA was 2.67 (P=0.025) when compared with the CC genotype. Further, our additive model revealed an association between the rs699947 polymorphism and collateral formation (OR: 1.96, 95% CI: 1.05–3.65, P=0.033).

**Conclusion:** The rs699947 polymorphism might be a novel genetic factor affecting collateral development in Iranian patients with coronary artery disease.

## Introduction

Collateral circulation improves the viability of the myocardium by supplying blood during hypoxemia.^1^^-^^3^ The formation of collaterals is a multifactorial process, and several determinants including genetic factors,^4^ the age of patients,^5^ and the severity of coronary artery disease (CAD)^6^ contribute to the formation of collaterals. Furthermore, hypercholesterolemia,^7^ systemic hypertension,^8^ a history of angina,^9^ cigarette smoking,^10^ diabetes mellitus,^11^ and certain kinds of medication,^10^ which have been implicated in the previous studies, are involved in the formation of collaterals. In this regard, some comprehensive studies have indicated that the levels of vascular endothelial growth factor (VEGF)^12^^, ^^13^ and endogenous angiogenesis inhibitors are involved in the formation of the collaterals.^10^^, ^^11^ These studies have shown that the VEGF is related to the development of atherosclerosis and lesion destabilization, although it is well known that the VEGF has some beneficial effects in the improvement of myocardial and peripheral ischemia.^14^ Certain recent experiments have proven that the atherosclerotic effect of the VEGF is a result of inflammatory plaque infiltration enhancement and neovascularization. These investigations have confirmed that the *VEGF* gene expression and the level of plasma VEGF in patients with CAD are significantly different from those in the normal population.^15^^-^^17^ In fact, in patients with CAD, the hypoxic situation produces hypoxia-inducible factor-1 (HIF-1), which regulates the expression of the *VEGF* gene and some other genes that are involved in adaptation to hypoxia, and results in angiogenesis via the activation of endothelial cell receptors.^18^^-^^23^ In this regard, it is well established that the *VEGF* polymorphism at the – rs699947 has functional significance; in patients with an A allele at the rs699947 location, the expression of the *VEGF* mRNA is considerably higher.^12^^-^^14^ To elucidate the effect of the VEGF on collaterals in patients with CAD, we performed the present study to evaluate the association between the VEGF plasma concentration and the *VEGF* rs699947 polymorphism and coronary collateral formation in patients with CAD, who had been referred to Tehran Heart Center.

## Methods

The study population comprised 195 consecutive patients scheduled for diagnostic coronary angiography between April 2010 and January 2011. Stable/unstable angina pectoris or any remote myocardial infarction was an indicator of catheterization. The criteria for enrollment were as follows: patient age >8 years and at least 1 coronary artery with stenosis ≥70%. Patients were excluded if they had anemia, acute myocardial infarction, and prior revascularization by percutaneous coronary intervention or coronary artery bypass graft surgery. In addition, subjects with clinical and laboratory features of acute or chronic inflammatory diseases, neoplastic diseases, and postmenopausal status, as well as women during menstrual cycles, were excluded because these conditions are known to affect the plasma VEGF.^24^^,^^25^ The study protocol was approved by the Ethics Committee of Tehran University of Medical Sciences.

The study was explained to the patients and their written informed letters of consent were collected. The enrolled patients were interviewed, and demographic information such as sex, age, birthplace, and profession was recorded. Moreover, the patients’ family history of CAD, diabetes, and smoking was recorded. Systolic and diastolic blood pressures were measured, and the left ventricular ejection fraction was assessed via echocardiography. Two reviewers, who were blinded to the study protocol and the biochemical measurements, analyzed each angiogram. The Rentrop scoring system was used to classify the coronary collateral circulation.^26^ A Rentrop score of 0 was given for no visible collateral, a score of 1 was given for visible collaterals without the dye reaching the epicardial segment of that vessel, a score of 2 was given for partial collateral filling of the epicardial segment of the vessel, and a score of 3 was given for the complete collateral filling of the vessel. In the case of discrepancies, a third reviewer, blinded to the readings of the first 2 reviewers, was requested to score the collateral formation. Finally, patients with collateral grades of 0 or 1 (poor collateral group; n=124) were compared with those with collateral scores of 2 or 3 (good collaterals group; n=71). Furthermore, the severity of coronary atherosclerosis was quantified using the Gensini score.^27^^, ^^28^

Two blood samples (each 2.5 cc) were taken from a femoral artery sheath placed for coronary artery catheterization and collected in ethylenediamine tetra-acetic acid tubes. One of the samples was immediately sent to the medical laboratory for VEGF assessment; the second was stored at –20 ^°^C until it was sent to the Cellular and Molecular Research Center, Ahvaz Jundishapur University of Medical Sciences, Ahvaz, Iran, for subsequent genotype analysis. Genotype analysis was conducted in an independent blinded manner by different members of the research team. A fasting serum sample was prepared for the measurement of lipids.

The serum levels of total cholesterol, high-density lipoprotein cholesterol, and triglyceride were determined by standard techniques using enzymatic methods. The level of low-density lipoprotein was measured based on the Friedewald formula.^29^ The serum level of high-density lipoprotein was evaluated in the supernatant after the precipitation of apolipoprotein B-containing lipoproteins by using phosphotungstic acid and magnesium chloride. Commercial kits *(Invitrogen, Camarillo,* *CA)* were used to measure the VEGF.

The rs699947 polymorphism, which is located in the promoter region of the *VEGF* gene, was genotyped by extracting genomic DNA from the buffy coat of the EDTA blood samples by using a standard salting out technique.^30^^, ^^31^ The presence or absence of the BglII restriction endonuclease recognition site within the *VEGF* gene was analyzed using the polymerase chain reaction-based restriction fragment length (PCR-RFLP). Oligonucleotide primers, used to determine the rs699947 polymorphisms, were the forward primer 5′-GGA TGG GGC TGA CTA GGT AAG C-3′ and the reverse primer 5′-AGC CCC CTT TTC CTC CAA C-3′. The PCR was carried out in a total volume of 20 μL. The reaction mixtures consisted of 100 ng of template DNA, 200 µM of dNTPs, and 0.2 µM of each primer as well as 1X PCR buffer and 1 unit of Taq DNA polymerase. At 95 ^°^C for 5 minutes, the PCR conditions were denaturized, followed by 32 cycles of denaturation at 95 ^°^C for 30 seconds; annealing was performed at 59 ^°^C for 30 seconds. To ensure a complete extension of all the PCR products, we performed the extension at 72 ^°^C for 50 seconds, followed by a final extension at 72 ^°^C for 10 minutes. The amplified PCR fragment of 324 bp was digested with the restriction enzyme BglII in a final appropriate digestion buffer at 37 ^°^C for at least 16 hours, followed by electrophoresis on 3% agarose gel. The substitution from a C to an A abolished a BglII cut site. The genotypes were termed “CC”, “CA”, or “AA”, where the A allele was coded for the presence of the BglII site and the C allele was coded for its absence. The homozygous variant CC produced 1 fragment of 324 bp, the homozygous variant AA produced 2 fragments of 202 bp and 122 bp, and the heterozygote CA produced 3 fragments of 324 bp, 202 bp, and 122 bp.

The data were entered in SPSS software, version 22. The *continuous* variables were presented as the mean±standard deviation (SD) and were summarized by numbers (%) for the categorical variables. The χ^2 ^test was used to compare the categorical data, and the independent 2-sample *t*-test or the Mann–Whitney test was employed to compare the *continuous* variables between the 2 groups. A multivariable regression model adjusted for sex, age, the number of diseased vessels, cigarette smoking, diabetes mellitus, and the use of anti-platelets and calcium-channel blockers and nitrate was established.

The correlation between the independent variables and collateral formation in the final model was presented as the odds ratio (OR) with a 95% confidence interval (CI). A 2-tailed P value < 0.05 was considered significant. The Hardy–Weinberg equilibrium was tested using the HWE program.

## Results

Among the 195 patients enrolled, 71 (36.4%) had collaterals. The univariate analysis of the variables between the groups showed that the good collateral group of patients had more diseased arteries (Table 1). The VEGF plasma levels did not show any significant differences between the patients with and without collateral vessels (P=0.637) (Table 2). 

**Figure 1:**
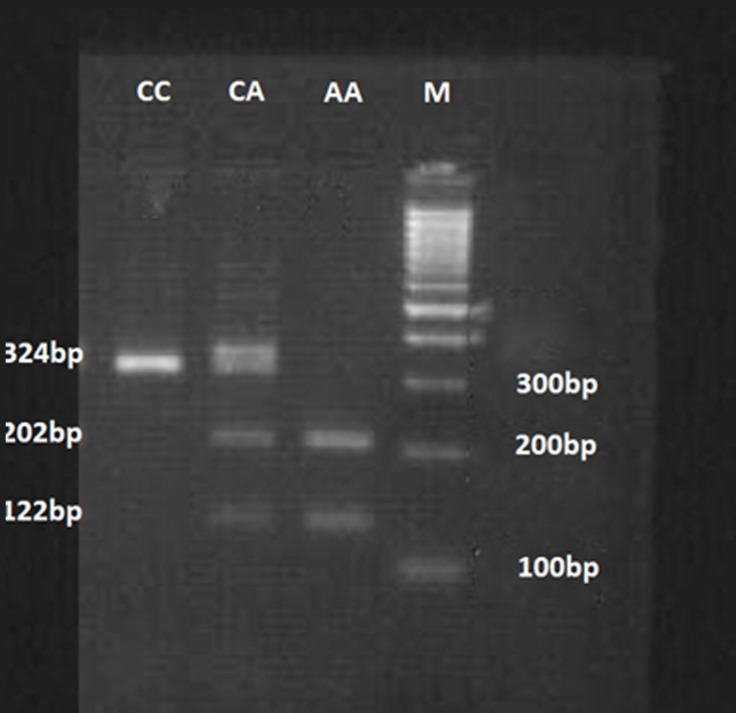
(PCR-RFLP analysis) Agarose gel (2%) electrophoresis of the BglII-digested PCR products is shown. A 324bp band for CC, 3 bands of 324, 202 and 122bp for CA, and 2 bands of 202 and 122bp bands for AA are shown. Lane M, 100bp DNA size marker.

For the polymorphism in the *VEGF* rs699947, the agarose gel electrophoresis of the BglII-digested PCR products is shown in Figure 1. The accuracy of the genotyping was confirmed by the sequencing of 10 randomly selected samples of the PCR products of the 3 variants. The patients were in the Hardy–Weinberg equilibrium for the rs699947 of the *VEGF* gene (HWE test: χ^2^=0.91, P=0.339). The frequency of the A allele was found to be higher in the patients with good collaterals than in those with poor collaterals (P=0.014). The OR of good collaterals for AA was 2.67 (P=0.025) when compared with the CC genotype. Our additive model demonstrated an association between the *VEGF* rs699947 polymorphism and collateral formation (OR: 1.96, 95% CI: 1.05–3.65, P=0.033).

PCR-RFLP, Polymerase chain reaction-restriction fragment length polymorphism

The multivariable analysis of the effect of the VEGF rs699947 genotypes on collateral formation in the patients with CAD adjusted for sex, age, the number of diseased vessels, cigarette smoking, and diabetes mellitus is shown in Table 3 (OR: 1.71, 95%CI: 0.87–3.39, P=0.122).

**Table 1:** Comparisons of the baseline characteristics between the poor and good collateral groups

	Collateral Grade										P value	
	0 (n=92)	1 (n=32)		2 (n=35)		3 (n=36)		Poor (n=124)		Good (n=71)	0-3	Good vs. Poor
Male	66 (71.7)	21 (65.6)	25 (71.4)		26 (72.2)		87 (70.2)		51 (71.8)		0.920	0.805
Age (y)	59.41±10.52	57.62±10.94	58.40±10.42		58.52±11.53		58.95±10.64		58.51±10.94		0.865	0.165
Diabetes mellitus	25 (27.2)	9 (28.1)	10 (28.6)		8 (22.2)		34 (27.4)		18 (25.4)		0.924	0.753
Hypertension	40 (43.5)	13 (40.6)	18 (51.4)		18 (50.0)		53 (42.7)		36 (50.7)		0.742	0.283
Smoking	40 (43.5)	13 (40.6)	13 (37.1)		8(22.2)		53 (42.7)		21 (29.6)		0.166	0.091
Family history of CAD	19 (21.1)	5 (16.1)	6 (18.2)		7 (21.2)		24 (19.8)		13 (19.7)		0.958	0.982
Hyperlipidemia	30 (32.6)	13 (40.6)	11 (31.4)		18 (50.0)		43 (34.7)		29 (40.8)		0.259	0.390
History of MI	20 (21.7)	12 (37.5)	6 (17.1)		7 (19.4)		32 (25.8)		13 (18.3)		0.187	0.232
Number of diseased vessels												
One	42 (45.7)	10 (31.3)	7 (20.0)		5 (13.9)		52 (41.9)		12 (16.9)		0.004	0.001
Two	28 (30.4)	8 (25.0)	14 (40.0)		12 (33.3)		36 (29.0)		26 (36.6)			
Three	22 (23.9)	14 (43.8)	14 (40.0)		19 (52.8)		36 (29.0)		33 (46.5)			
BMI (kg/m^2^)	25.72±4.19	26.41±3.39	27.17±4.00		27.80±3.27		26.65±3.99		27.48±3.64		0.477	0.781
Medication												
Antiplatelet agent	55 (59.8)	14 (43.8)	9 (25.7)		17 (47.2)		66 (53.2)		26 (36.6)		0.006	0.029
Beta-blocker	82 (89.1)	27 (84.4)	31 (88.6)		32 (88.9)		109 (87.9)		63 (88.7)		0.891	0.863
Nitrate	81 (89.0)	27 (87.1)	27 (79.4)		36 (88.9)		108(88.5)		54 (79.4)		0.399	0.089
ACEI or ARB	31 (33.7)	14 (43.8)	10 (28.6)		11 (30.6)		45 (36.3)		21 (29.6)		0.570	0.340
Calcium-channel blockers	27 (29.3)	8 (25.0)	12 (34.3)		15 (41.7)		35 (28.2)		27 (38.0)		0.448	0.157
Diuretic	6 (6.5)	3 (9.4)	1 (2.9)		2 (5.6)		9 (7.3)		3 (4.3)		0.757	0.541
Statin	67 (72.8)	25 (78.1)	19 (54.3)		29 (80.6)		92 (74.2)		48 (67.6)		0.062	0.325

^*^Data are presented as mean±SD or number (%).

Comparison of age and the BMI across collateral grades was performed using the analysis of variance test, and the comparison of those between the good and poor collateral groups was performed using the independent 2-sample* t*-test. Comparison of the categorical variables was done by using the Pearson χ^2^ test.

CAD, Coronary artery disease; MI, Myocardial infarction; BMI, Body mass index; ACEI, Angiotensin-converting enzyme inhibitors; ARB, Angiotensin receptor blockers

**Table 2:** Comparison of the vascular endothelial growth factor )VEGF( concentration and the VEGF −2578 genotype between the poor and good collateral groups

	Collateral Grade							P value	
	0 (n=92)	1 (n=32)	2 (n=35)	3 (n=36)	Poor (n=124)	Good (n=71)	OR (95% CI)	0-3	Good vs. Poor
VEGF Concentration (pg/mL)*	142.82 (43.3-269.7)	164.53 (70.3- 290.5)	188.41 (77.9- 504.5)	92.21 (37.3-418.2)	152.52 (49.8-271.6)	163.54 (41.1-432.5)	-	0.284	0.637
Allele frequency								0.015	
C	117 (63.6)	47 (73.4)	42 (60.0)	34 (47.2)	164 (66.1)	76 (53.5)	1		0.014
A	67 (36.4)	17 (26.6)	28 (40.0)	38 (52.8)	84 (33.9)	66 (46.5)	1.69 (1.11-2.58)		
Genotype								0.098	
CC	39 (42.4)	17 (53.1)	14 (40.0)	7 (19.4)	56 (45.2)	21 (29.6)	1		0.062
CA	39 (42.4)	13 (40.6)	14 (40.0)	20 (55.6)	52 (41.9)	34 (47.9)	1.74 (0.90-3.38)		0.100
AA	14 (15.2)	2 (6.3)	7 (20.0)	9 (25.0)	16 (12.9)	16 (22.5)	2.67 (1.13-6.29)		0.025
Additive model for A allele effect							1.96 (1.05-3.65)		0.033

Comparison of the VEGF concentration across the collateral grades was performed using the analysis of variance test, and the comparison of that between the good and poor collateral groups was performed using the Mann–Whitney *U*-test. The categorical variables were compared using the Pearson χ^2^ test.

* Values are presented as median (IQR_25–75%_) or n (%).

**Table 3:** Unadjusted and adjusted effects of the vascular endothelial growth factor (VEGF) genotype on collateral formation in the patients with coronary artery disease

	OR	95% CI	P value
Unadjusted effect of VEGF -2578 C > A SNP (CC as reference)	1.96	1.05-3.65	0.033
Adjusted* effect of VEGF -2578 C > A SNP (CC as reference)	1.71	0.87-3.39	0.124

Single-nucleotide polymorphism

* Adjusted for sex; age; the number of diseased vessels; cigarette smoking; diabetes mellitus; and the use of antiplatelets, calcium-channel blockers, and nitrate

## Discussion

Angiogenesis is an important factor in the development of coronary collateral vessels.^32^ It is well recognized that the VEGF plays an important role in the process and the cardiac VEGF level is related to coronary collateral formation.^33^ Although the VEGF has been considered a therapeutic target in patients with CAD,^34^ the circulating VEGF concentration in patients with CAD has proven to be a controversial subject.^16^^, ^^17^^, ^^35^ Additionally, a number of studies have reported that the plasma level of the VEGF is higher in the good collateral group and lower in the poor collateral group of patients with CAD; however, these differences are not statistically significant.^36^^-^^39^

In our study, the intra-patient variations of the VEGF plasma concentration within the poor and good collateral patient groups were large and no significant differences were found in the plasma VEGF concentrations between the groups. The exogenous and endogenous inhibitors of the VEGF may explain the wide variation in the plasma VEGF in patients with CAD. A number of common medications have been identified that may interfere with angiogenesis; these include common heart disease drugs and certain important noncardiac drugs. Other confounding factors including age, hypercholesterolemia, smoking, and diabetes have also been suggested.^10^

The rs699947 is located in the promoter region of the *VEGF* gene on the chromosome 6p21.3. The presence of the A allele of rs699947 is associated with an increased VEGF expression.^40^ In the current study, the frequency of the A allele of the *VEGF* rs699947 was lower than that of the C allele among all our patients with CAD. However, the distribution of the A allele among the patients with good collaterals was significantly higher than that among those with poor collaterals (P=0.014). Several studies have reported allele and genotype distributions similar to the findings in our study.^41^^-^^44^ We also found evidence indicating a possible association between the AA genotype of rs699947 and collateral formation (OR: 2.67, 95%CI: 1.13–6.29, P=0.025) and in the additive model for the effect of the A allele (OR: 1.96, 95%CI: 1.05–3.65, P=0.033). Although after we made adjustments for potential confounders (sex, age, the number of diseased vessels, cigarette smoking, and diabetes mellitus), the P value was at the margin of statistical significance (OR: 1.71, 95% CI: 0.87–3.39, P=0.122); our findings represent sufficient evidence to suggest the effect of the rs699947 polymorphism on adaptive collateralization in patients who have CAD. This finding is consistent with several reports showing that the A allele is a positive independent predictor of angiogenesis.^42^^-^^45^ Well-developed collaterals may help protect the myocardium during a hypoxic situation. Thus, individual differences in collateral development may be considered a reliable indicator of cardiac vulnerability. 

Our findings suggest that variations in the *VEGF* gene might partly account for differences in collateral development. There are several factors other than the growth factors that may affect collateral formation. In the current study, there was a strong association between the number of diseased vessels and coronary collateral development (P=0.001). Our results are consistent with the findings of previous studies, which have identified the number of diseased coronary vessels as the promoting factor of collateral formation.^6^^, ^^10^^, ^^46^^-^^48^ 

## Conclusion

In conclusion, we showed that the *VEGF* plasma concentrations did not correlate with collateral development in CAD patients. We demonstrated that the *VEGF* rs699947 polymorphism might influence collateral formation in CAD patients and contribute to the differences between individuals’ susceptibilities to ischemia.

The current study has several limitations. First, the sample size was small and the patients were enrolled from a single center. However, it is important to perform larger multicenter studies with a larger statistical power to better elucidate the role of this polymorphism in patients suffering from CAD. Second, other *VEGF *polymorphisms may affect the VEGF concentration and function. Thus, further investigations on functional *VEGF* polymorphisms and haplotypes are required to confirm these findings. Finally, with regard to the presence of considerable challenges in the assessment of collateral vessels using angiography, the use of more accurate techniques such as micro-CT analysis, magnetic resonance angiography, and 3D reconstruction of tomographic images is suggested for future studies.

## Notes:

***This paper should be cited as: ***Alidoosti M, Shanaki M, Mahdavi A, Mohammadtaghvaei N. Association between Vascular Endothelial Growth Factor Plasma Levels and rs699947 Polymorphism and Coronary Collateral Vessel Formation. J Teh Univ Heart Ctr 2019;14(3):121-127.
